# Ultraviolet photolysis of 1,2-dimethyldisilane in the gas phase

**DOI:** 10.1016/j.dib.2018.08.074

**Published:** 2018-08-29

**Authors:** Najem Al-Rubaiey

**Affiliations:** Petroleum Technology Department, University of Technology, Baghdad, Iraq

## Abstract

The formation of MeSiH is the primary process in the photolysis of 1,2-dimethyldisilane at 193 nm that are analogues of carbenes. Gas chromatographic technique was used with a flame ionization detector as an analysis tool to identify the products mixture. The photolysis light at 193 nm was provided by an Oxford KX2 pulsed laser operated with rare-gas halide (known as an excimer laser) as the gain medium to provide ultraviolet (UV) radiation. This work has confirmed that radical processes are not important in the photolysis of 1,2-dimethyldisilane. A method for the determination of rate constants for MeSiH reactions relative to the rate constants of 1,2-dimethyldisilane has been formulated. This has been used to determine some relative rate constants of MeSiH insertions with methylsilanes. The insertion reactions of MeSiH with SiH_4_ and Methysilanes have shown to be fast and closer in reactivity to SiH_2_ than to SiMe_2_, whereas PhSiH looks to be slightly more reactive than MeSiH.

**Specifications Table**

**Table:** 

Subject area	*Chemistry*
More specific subject area	*Photochemistry*
Type of data	*Table, figure, scheme*
How data was acquired	*Experimental tests*
Data format	*Analyzed*
Experimental factors	*None*
Experimental features	*End product technique using gas chromatography for analysis and UV laser source as a photolysis tool*
Data source location	*Reading, UK*
Data accessibility	http://ethos.bl.uk/OrderDetails.do?uin=uk.bl.ethos.283700
Related research article	*Najem Al-Rubaiey, Kinetic investigation of some prototype silylene reactions induced by laser flash photolysis, Doctoral Thesis, University of Reading, Reading, UK, 1994, ISNI: 0000 0001 3408 1417.*

**Value of the data**

• Silylene can be considered as very reactive intermediates. They have a very important role in the chemical vapor deposition of many thin films that contain silicon compounds. These species have technological importance in microelectronics industry besides the dry etching process of silicon wafers.
• The result presented here shows that MeSiH is much closer in reactivity to SiH_2_ than to SiMe_2_ whereas PhSiH looks to be slightly more reactive than MeSiH.
• This work also suggests that the reaction will happen in two stages, the first "electrophilic" stage in which the electrons from the Si–H bond feeds into the empty p orbital on the silylene, this follows by the second "nucleophilic" stage which corresponds to donation of the lone-pair on the silylene to make new Si–Si bond.

## 1 Data

DMDS photochemistry was explored by investigating the experimental effects of the number of laser shots, DMDS pressure, total pressure, added oxygen and temperature. In order to assess the effects, The GC peaks ratio [MeSiH_3_]/[DMDS] was measured. This ratio gives an indication of the photochemical conversion. TMTS was not identified (and therefore not monitored) at the beginning of the work. A number of points can be extracted from this study:

1) The results show the linear dependence of the ratio with the number of shots. This suggests a well behavior reaction with product yields proportional to absorbed photons.
2) It demonstrates that the more DMDS in the cell, the less of [MeSiH_3_]/[DMDS] ratio. A possible explanation is that most of the 193 nm radiation light is absorbed by the DMDS at the front end of the cell. Therefore, the more DMDS in the cell the more MeSH_3_ formed is diluted in unphotolysed DMDS. Another possible explanation is related to the film build up which has similar effect by reducing the amount of radiation going through the cell. To minimize difficulties of high light absorption or film formation, the pressure of DMDS was kept as low as possible. In practice the pressure range was 50–100 mTorr.
3) The results confirm that the ratio [MeSiH_3_]/[DMDS] did not vary beyond experimental error, with change in total pressure. This suggests that the reaction is pressure independent within the scatter.
4) The results show that adding oxygen does not affect the ratio of [MeSiH_3_]/[DMDS] within experimental error which suggests that the primary process is not radical process but rather molecular extrusion process.
5) In addition to MeSiH_3_, TMTS, Me_2_SiH_2_ and SiH_4_, no other products were detected at 298 K or higher temperature.

Another set of experiments was carried out with added SiH_4_, Me_2_SiH_2_, and Me_3_SiH in order to confirm the production of MeSiH (by scavenging it *via.* Si-H insertion processes). When DMDS is photolysed in the presence of added gas then the latter will compete with the unphotolysed DMDS to trap the methylsilylene formed. A depletion in the amount of TMTS will be observed, and thus will be an increase in the ratio of MS to TMTS. The first group of experiments with silane, dimethylsilane and trimethylsilane (which do not absorb significantly at 193 nm) were carried out at room temperature. The linear dependence of the product ratios on substrate pressures (relative to DMDS) in all cases shows that these results confirm to the relationship determined here. Applying the relative rate procedure described previously, the relative rate constants can now be calculated with two figures for each reaction.

## 2 Experimental design, materials, and methods

A Pyrex glass, static, high vacuum line system has been used. The line was pumped by a mercury diffusion pump backed by a single stage rotary oil pump. Gas chromatographs were used for the analysis. Several types of columns were employed for analytical purposes, the packing materials and operating conditions of which were dependent on the reaction under investigation. Five main cylindrical reaction cells were used for the kinetic runs. The photolysis light was provided by an Oxford KX2 pulsed laser operated with rare-gas halid as the gain medium to provide ultraviolet radiation. Samples for photolysis were prepared by putting the required pressure of precursor into the reaction cell then adding the other reacting substrate (if required) and finally making the reaction mixture up with nitrogen to the required pressure. After the photolysis, the mixture of gases in the cell was analyzed by gas chromatography. The procedure for a particular substrate gas was to vary the ratio of precursor to the gas added and monitor the change in the ratio of products produced by gas chromatography.
