# Supplementary material for: Ultraviolet photolysis of 1,2-dimethyldisilane in the gas phase
Source: Data Brief. 2018 Aug 29;23:102962. doi: 10.1016/j.dib.2018.08.074 (PMC6657231; doi:10.1016/j.dib.2018.08.074)
Supplement: Supplementary file 1 — Supplementary material [file mmc1.docx]

**Data** [**Supplementary**](https://www.google.iq/search?rlz=1C1GGRV_enIQ752IQ752&q=supplementary&spell=1&sa=X&ved=0ahUKEwiVzILWvoLaAhVHXSwKHVZ4DVcQkeECCCIoAA)**:**

**Appendix:**

Figure 1. Dependence of [MeSiH_3_]/[DMDS] on number of shots.

Figure 2. Dependence of [MeSiH_3_]/[DMDS] on DMDS pressure.

Figure 3. Dependence of [MeSiH_3]_/[DMDS] on total pressure.

Figure 4. Dependence of [MeSiH_3_]/[DMDS] on oxygen.

Figure 5. Dependence of [MeSiH_3_]/[DMDS] on temperature.


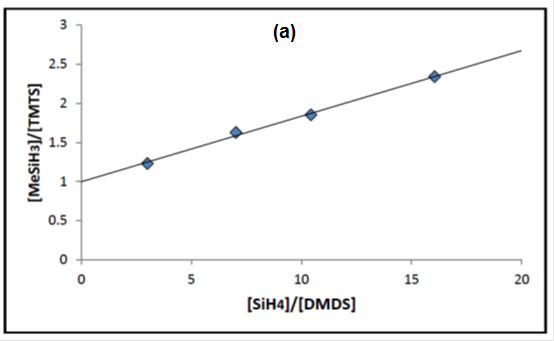


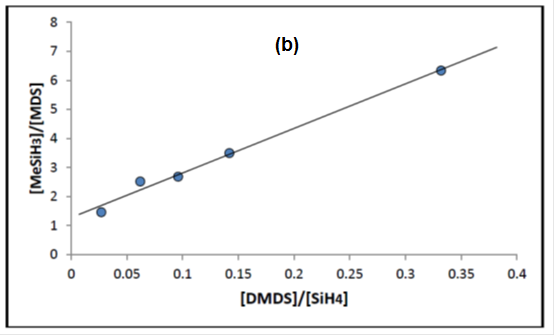


Figure 6. Dependence of product on added substrate: DMDS + SiH_4_


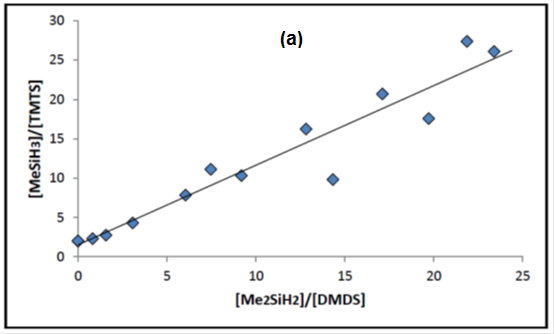


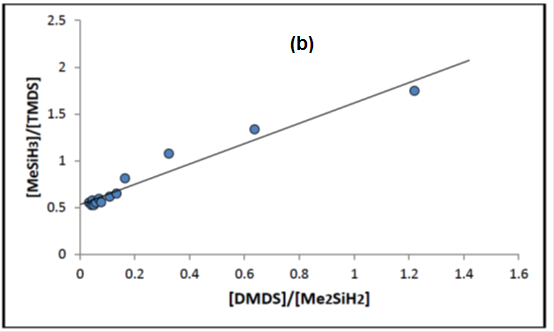
Figure 7. Dependence of product on added substrate: DMDS + Me_2_SiH_2_


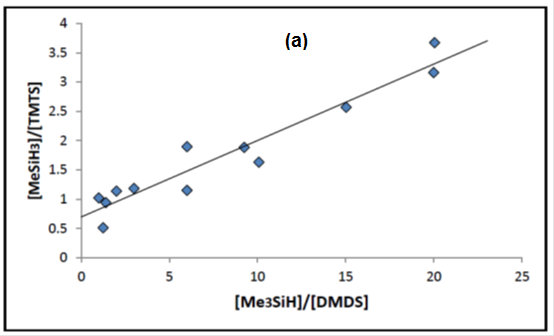


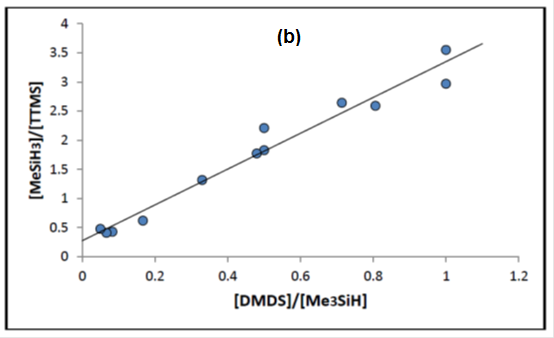
Figure 8. Dependence of product on added substrate: DMDS + Me_3_SiH

Scheme 1. Nature of the silylene insertion process.

Scheme 2. Silylene insertion: intermediate complex[22].

Table 1. Relative rate constants of methylsilylene removal by silane, dimethylsilane and trimethylsilane at 298K (from two different processes).

| **Substrate** | **(k_x_/k_DMDS_)_1_** | **(k_x_/k_DMDS_)_2_** |
| --- | --- | --- |
| SiH_4_ | 0.08 ± 0.01 | 0.08 ± 0.01 |
| Me_2_SiH_2_ | 0.66 ± 0.47 | 0.49 ± 0.09 |
| Me_3_SiH | 0.19 ± 0.04 | 0.09 ± 0.04 |

Table 2. Absolute rate constants comparison for thee reaction of MeSiH with methylsilanes at 298K and 200 Torr total pressure.

| **Substance** | **k/ 10^-11^ cm^3^ molecule^-1^ s^-1^** | | |
| --- | --- | --- | --- |
|  | **Becerra *et al.* [6]** | | ***This work*** |
|  | **D (1)** | **D (2)** |  |
| SiH_4_ | 8.1 | 12.9 | 3.97 |
| MeSiH_3_ | 25.5 | 19.5 | - |
| Me_2_SiH_2_ | 16.9 | 25.5 | 23.18 |
| Me_3_SiH | 27.8 | 16.3 | 6.62 |

Table 3. Absolute rate constant at 298K, k/10^-11^ cm^3^ molecule^-1^ s^-1^.

| **Substrate** | **SiH_2_[17]** | **SiMe_2_[18]** | **MeSiH** | **PhSiH[19]** |
| --- | --- | --- | --- | --- |
| SiH_4_ | 42 | 0.02 | 3.97 | 5.1 |
| MeSiH_3_ | 37 | 0.19 | - | 39 |
| Me_2_SiH_2_ | 33 | 0.55 | 23.18 | 21 |
| Me_3_SiH | 25 | 0.45 | 6.62 | 39 |
